# The Effectiveness of Mind-Body Intervention on Psychological Well-Being during the COVID-19 Pandemic: A Pilot Pre-Post Interventional Study

**DOI:** 10.3390/healthcare12111125

**Published:** 2024-05-31

**Authors:** Aaron Peterson, Philip Borsellino, Ryder Davidson, Edozie Ezeanolue, Gemma Lagasca, Jared Diaz, Kavita Batra, Anne Weisman

**Affiliations:** 1 Kirk Kerkorian School of Medicine at UNLV, University of Nevada, 625 Shadow Lane, Las Vegas, NV 89106, USA; borsep1@unlv.nevada.edu (P.B.); davidr6@unlv.nevada.edu (R.D.); ezeane2@unlv.nevada.edu (E.E.); lagasg1@unlv.nevada.edu (G.L.); diazj@unlv.nevada.edu (J.D.); 2 Department of Medical Education, Kirk Kerkorian School of Medicine at UNLV, University of Nevada, 625 Shadow Lane, Las Vegas, NV 89106, USA; 3 Office of Research, Kirk Kerkorian School of Medicine at UNLV, University of Nevada, 1701 West Charleston Blvd, Las Vegas, NV 89102, USA

**Keywords:** mind-body medicine, COVID-19, depression, pandemic, virtual, integrative

## Abstract

The pandemic highlighted the need for alternative, more accessible access to mental health interventions that can be readily administered remotely. The purpose of this pre-post-interventional study was to evaluate the effectiveness of a virtual mind-body medicine training course on stress, anxiety, and depression levels. University employees and members of the Las Vegas community were recruited via self-selection and snowball sampling and subjected to online mind-body practice sessions in December of 2020. Stress, anxiety, depression, and quality of life were assessed pre- and post-intervention using standardized psychometric valid tools. The paired t-test and related samples marginal homogeneity tests were used for continuous and categorical outcomes, respectively. Depression and stress scores were significantly decreased (*p* < 0.001). Mean scores of professional quality of life improved post-intervention compared to pre-intervention (*p* = 0.03). A significantly larger proportion of participants reported no depression or stress post-intervention compared with pre-intervention (*p* < 0.001, *p* = 0.003, respectively.) This study suggests that virtual mind-body practices had a pronounced impact on stress and depression levels during the pandemic. These findings support virtual, online-guided mind-body medicine training as an effective intervention that can be administered virtually to reduce stress and depression symptoms.

## 1. Introduction

In 2020, the Severe Acute Respiratory Syndrome-2 (SARS-CoV-2) virus triggered a worldwide pandemic that resulted in lockdowns in nearly all countries. Due to theCOVID-19 pandemic-related social distancing and quarantine measures, normal socialization was disrupted. Subsequent isolation, uncertainty, fundamental changes in societal norms, and limited medical resources all contributed to increased stress levels during the pandemic [1]. Responses to stressors during the pandemic varied among individuals, with an increased prevalence of mental health disorders and maladaptive responses documented [1,2,3,4].

During the initial stages of the COVID-19 pandemic and the ensuing lockdowns, there was a marked increase in stress levels that coincided with heightened substance use [1,2,3,4]. This period also saw an associated rise in overall drug overdose incidents and reports of suicidal ideation [5,6,7]. While alcohol use demonstrated an overall increased trend, increases in other substance use during the pandemic demonstrated a strong positive trend in the general population [8]. Although it has been demonstrated that stress is positively associated with motivation for the use of substances as a coping strategy, causality has yet to be definitively established [8]. Furthermore, substance misuse is positively associated with mood and anxiety disorders, such as depression and post-traumatic stress disorder (PTSD) [8,9].

The COVID-19 pandemic precipitated an upsurge in mental health disorders worldwide, with a 27.6% increase in major depressive disorders and a 25.6% escalation in anxiety disorders throughout 2020 [5]. Although the pandemic affected the majority of the population, the severity of deleterious psychological symptoms, such as stress, anxiety, isolation, and depression varied among population subgroups. Some of the most affected groups were patients and frontline workers. The prevalence of anxiety and sleep disturbances in COVID-19 patients increased when compared to the general population [6]. Psychological symptoms represented among healthcare workers were notably intensified in nurses, frontline workers, and females [7]. As a result, frontline workers with increased exposure to COVID-19 were particularly susceptible to developing professional burnout and compassion fatigue when appropriate interventions to support mental well-being were not taken [8].

A meta-analysis of longitudinal cohort studies revealed an acute surge in mental health symptoms at the pandemic’s inception [5]. However, these symptoms exhibited a significant decline over time, ultimately becoming indistinguishable from pre-pandemic symptom profiles within a few months of the outbreak. These results suggest normal response and adaptation to acute external stress [5]. However, high levels of stress associated with COVID-19 are concerning, and long-term sequelae from chronic stress must also be monitored and mitigated. Chronic stress elevation may lead to the onset of psychological comorbidities, such as anxiety, depression, and substance abuse, or physical comorbidities, including the exacerbation of existing conditions [9]. Therefore, despite the reduction in stress back to pre-pandemic levels, the increase in prevalence of disorders related to stress can have long-lasting consequences. The increase in these problems underscores the need for effective interventions to address the social and psychological consequences of the COVID-19 pandemic and lockdown. This further underscores the critical need for timely and effective mental health interventions during times of crisis or pandemics in order to foster resilience and expedite post-traumatic recovery among affected population groups.

Psychological/psychiatric interventions have been recommended to promote mental health, with individual psychotherapies commonly recommended. Integrative or non-traditional therapies were also recommended, with meditation, yoga, and breathing techniques being the most common interventions [10]. Meditation was found to have a positive effect on mood and anxiety for individuals in stressful situations including COVID-19, and was found to have induced neurobiological changes within 8-week programs [11].

Research into the benefits of integrative therapies, including mind-body medicine, has grown significantly. Specifically, mind-body medicine is an integrative health practice utilizing techniques including meditation, breathing techniques, biofeedback, or combination therapies to help relax the body and mind, and to lower pain, stress, anxiety, and depression [12]. A systematic review of randomized control trials by Duong et al. demonstrated that treatment with multiple mind-body medicine interventions significantly reduced cancer-related fatigue, with meditation and mindfulness interventions contributing the greatest effect [13]. The use of mind-body group therapy has also been shown to decrease the severity of chronic depression, with a systematic review by D’Silva finding that several evidence-supported mind-body medicine techniques show efficacy in lowering depression severity [14,15]. Current evidence supports the integration of integrative medicine treatments as adjunct therapy to currently established psychiatric treatments for depression, among other psychiatric conditions.

A systematic review examined the effectiveness of various mental health interventions, including crisis intervention sessions, mobile phone-delivered interventions, music therapy, phone consultations, progressive muscle relaxation, internet-based self-help intervention, and mind-body interventions [16]. Specifically, the review focused on the effects of these interventions during the COVID-19 pandemic, and found that the non-psychiatric mental health interventions were effective in addressing mental health concerns during both the COVID-19 pandemic and other medical pandemics [16].

### About the Program/Intervention

To address the mental health challenges brought on by the pandemic, the Center for Mind-Body Medicine (CMBM) in partnership with the Kirk Kerkorian School of Medicine at UNLV and School of Public Health at University of Las Vegas (UNLV) proposed a mind-body medicine training program for UNLV staff, faculty, and students, as well as targeted community members and service providers. CMBM’s 5-day virtual training program comprised a blend of didactic instruction and large and small group experiences of evidence-based mind-body techniques. The group experience was critical to the training, and participants were encouraged to participate fully in each session. Participants were introduced to mind-body medicine, the physiology of stress, and different techniques for self-regulation, including breathing, meditation, movement, mindfulness, writing exercises, visualization, guided imagery, and autogenic training. The exercises were taught and experienced in large and small groups, with sharing encouraged.

The training began with a live presentation about mind-body medicine along with an experiential exercise. Unlike other traditional training programs, this program was designed to be highly experiential. In other words, participants learned and were taught about the techniques and the model through their own first-hand experience of the techniques and the model, which could be further shared with the other community members.

Over the course of five days, participants received didactic instruction on the scientific basis for CMBM’s model and methods. Following topics such as the biology and psychology of stress and trauma, discussion on the evidence related to the CMBM approach, extensive instruction (experiential and didactic) on foundational evidence-based skills of self-care, and self-awareness were covered. Following this, participants were assigned to a small group that would meet two times a day throughout the training, with the exception of the one shorter training date, in which only one small group session was held. The small groups were held so that participants could deepen their knowledge and understanding of the material and how it impacted them. These group discussions in the advanced training phase allowed participants to use and to share the techniques and model with their communities. The training followed a schedule that was communicated in advance for the participants, as attendance and full participation were necessary.

The proposed program served as a pilot project, laying the foundation for a future innovative regional healing and well-being program. The CMBM has developed this program over the last 30 years, which has been proven to reduce diagnosable PTSD by 80% and enhance mood and decrease anger and hopelessness [17,18,19]. This initiative has been used in a variety of contexts, including post-conflict, natural disasters, school shootings, the opioid crisis, among indigenous groups, and with active-duty military personnel, veterans, and their families. The program has been successful in bringing together many individuals, including those who may have been angry at and fearful of one another [20]. With its evidence-based approach, the CMBM program has been shown to positively influence mental health and well-being, and this pilot offers a chance to see how well it copes with the specific challenges posed by the pandemic and current events. Therefore, the purpose of this pilot study was to evaluate the impact of the CMBM program on addressing the mental health difficulties brought on by the COVID-19 pandemic on a majority-female community population.

## 2. Methods

### 2.1. Study Design, Setting, and Participants

This pilot interventional study utilized a pre-post design, and the sample constituted UNLV students, faculty, and staff; UNR students, faculty, and staff; members of the Las Vegas community; and members of the Reno community. The demographic characteristics of the study population (n = 94) were as follows: the median age was 45, with an interquartile range of 20 years, and the gender distribution was 87.2% female (n = 82) and 12.8% male (n = 12). Ethnically, 4.3% were Hispanic (n = 4), 61.7% were non-Hispanic (n = 58), and 34.0% did not report their ethnicity (n = 32). Regarding race, 52.1% were white (n = 49), 8.5% were non-white (n = 9), and 38.3% did not report their race (n = 36). The roles of the participants were primarily staff (64.9%, n = 61) and community members (35.1%, n = 33). This study was conducted in December 2020. Participants engaged in virtual mind-body medicine training led by CMBM faculty, with the program’s effectiveness measured using various scales for depression, anxiety, and stress.

### 2.2. Ethical Considerations

The following study was approved by the UNLV Biomedical Institutional Review Board (IRB-1683905-3) and received an exempt status. Participation was completely voluntary and detailed information about the intervention and study were provided to the participants. Informed consent was obtained.

### 2.3. Sample Recruitment

Ninety-four participants in this study were recruited through email, word of mouth, and institutional listserv. Participants were selected based on self-selection and snowball sampling. All subjects were over 18 years old and included faculty, staff, and leadership of UNLV, and members of the Las Vegas community, the Reno area, and UNR. In Reno specifically, students, faculty, and staff were recruited, but primarily staff and faculty participated. After informed consent was obtained, pre-assessments were obtained using the Depression, Anxiety, and Stress Scale (DASS-21), the Professional Quality of Life Scale (for faculty only), and the Social Connectedness Scale, respectively, via the Qualtrics survey tool.

### 2.4. Sample Justification

Given the pilot nature of this interventional study, we relied on the conventional method of power analysis. G power software (version 3.1) was used to perform a priori power analysis [21,22,23]. Given the repeated measures, pre-post design, effect size of 0.50, and a power of at least 80% at 5% alpha error probability, a minimum sample size of N = 34 was deemed appropriate.

### 2.5. Instruments

To measure the effectiveness of the program, three scales were used: the DASS-21 Scale, Professional Quality of Life Scale, and Social Connectedness Scale.

The Depression, Anxiety, and Stress Scale (DASS-21) is a self-report questionnaire used to assess symptoms of depression, anxiety, and stress in adults. This tool has excellent internal reliability, demonstrated by a Cronbach alpha of 0.74 and an ordinal alpha for the subsections, DASS-D (depression), DASS-A (anxiety), and DASS-S (stress), which were 0.83, 0.74, and 0.87 respectively [24]. There are 21 items on the scale that measure the severity of stress, anxiety, and depressive symptoms. Participants were asked to rate the frequency of each symptom on a four-point scale, from “never” to “often”.

The Professional Quality of Life Scale (ProQoL) assesses the physical and emotional well-being of healthcare workers. Because it is designed for academic staff members, this scale was only completed by UNLV faculty participants and UNR participants. There are 30 items on the scale that assess the psychological and physical well-being of professionals in the areas of secondary traumatic stress, compassion fatigue, and satisfaction with one’s capacity for helping others. Participants were asked to rate the frequency of each symptom on a five-point scale, from “never” to “often” [24].

The Social Connectivity Scale (SCS) measures how socially connected a person feels. There are 20 items on this scale that measure a person’s sense of connection to others, comfort in expressing oneself to others, and sense of support from others. Participants were asked to rate the frequency of each symptom on a five-point scale, from “never” to “often” [25]. Please see Table 1, which describes scoring criteria, measurement levels, cut-offs, and reliability diagnostics.

### 2.6. Procedure

All surveys were conducted electronically using Qualtrics. Participants were randomly assigned a number and took the assessments using this number. Only the PI and research team had access to the data.

The study was divided into three periods: the pre-observation period, the pilot-observation period, and the post-observation period.

In the pre-observation period, participants completed the Depression, Anxiety, and Stress Scale (DASS-21), the Professional Quality of Life Scale (for faculty only), and the Social Connectedness Scale electronically.

In the pilot-observation period, participants completed a virtual 5-day mind-body medicine training led by faculty from The Center for Mind-Body Medicine. At the conclusion of the 5-day training, participants again completed the DASS-21 Scale, the Professional Quality of Life Scale (for faculty only), and the Social Connectedness Scale electronically.

In the post-observation period, data were analyzed, and participants were followed-up one-week post-intervention. The scales were repeated once more electronically.

### 2.7. Data Analysis

First, univariate, and bivariate tests were conducted to analyze the data. Categorical variables were reported as frequencies or percentages. Continuous variables were presented as mean and standard deviation if normally distributed. For assessing the normality assumption, we used the Shapiro–Wilk test. For some of the outcome variables, the results of the Shapiro–Wilk test were significant, which was indicative of a non-normal distribution. Therefore, we applied transformation to the data for the normal approximation. Pre- and post-mean scores of psychological outcomes were compared using a paired *t*-test, while categorical outcomes were compared using related-samples marginal homogeneity tests. A Pearson correlation test was also utilized to ascertain relationships between psychological outcomes and professional quality of life. The significance level was set at 5% and the normal approximation to the binomial distribution method was used to calculate 95% confidence intervals of proportions in the univariate analyses. All analyses were conducted using SPSS version 28.

## 3. Results

### 3.1. Univariate Analysis

In a total sample of 94 program participants, over 85% were females and over 50% were white (Table 2). The median age of the sample was 45 years (IQR = 20 years). Most of the participants were from the UNLV staff/faculty, while over 30% were community members.

### 3.2. Bivariate Analysis

The results of the paired *t*-test indicated that there were statistically significant differences in the mean scores of depression (5.60 ± 4.72 vs. 2.37 ± 2.77, *p* < 0.001), stress (10.3 ± 6.69 vs. 2.82 ± 1.30, *p* < 0.001), and overall DASS (21.30 ± 13.89 vs. 5.64 ± 4.89, *p* < 0.001) at pre- vs. post-intervention periods (Table 3). The mean scores of professional quality of life were improved post-intervention as opposed to pre-intervention levels (82.97 ± 8.07 vs. 80.89 ± 7.57, *p* = 0.03). There were no statistically significant differences noted between pre- and post-interventional mean scores of anxiety, compassion satisfaction, burnout, secondary traumatic stress, and social connectedness (*p* > 0.05, Table 3).

As revealed by the Pearson correlational analyses, depression was directly and moderately correlated with anxiety (r = 0.430, *p* < 0.01) and stress (r = 0.562, *p* < 0.01), and weakly correlated with burnout (r= 0.211, *p* < 0.01, Table 4). Depression was negatively and weakly correlated with compassion satisfaction (r = −0.247, *p* < 0.01). Burnout was negatively and moderately correlated with compassion satisfaction (r = −0.689, *p* < 0.01). However, burnout was directly and moderately correlated with secondary traumatic stress (r = 0.420, *p* < 0.01, Table 4).

Upon comparing levels of depression, a significantly larger proportion of participants reported no depression post-intervention (85.1%) as opposed to pre-intervention (60.6%, *p* < 0.001). Similarly for stress levels, a significantly larger proportion of participants reported no stress post-intervention (89.4%) as opposed to pre-intervention (61.7%, *p* = 0.003, Table 5).

## 4. Discussion

The decrease in stress and depression symptoms among participants supports the efficacy of the novel delivery method of a virtual, online-guided mind-body medicine program. Benefits to virtual delivery methods include a decreased transportation burden, lower cost, and potentially, the recruitment of a larger number of participants. Downsides to virtual programs include technical difficulties, lack of interpersonal interaction, and lack of technological literacy among participants [25,26]. Future studies will be necessary to determine the relative effectiveness of virtual, online-guided interventions as compared to traditional in-person interventions; however, such interventions represent a promising alternative or adjunctive to other in-person, non-medical modalities.

Although there was a marked reduction in stress and depression among study participants, it is unclear why there was no significant reduction in participants’ anxiety or in anxiety scores post-intervention. Large systematic reviews have previously shown significant reductions in anxiety within certain patient populations, and modest to mixed effects in patients with generalized anxiety disorder [26,27,28,29]. Failure to show significant effects on anxiety levels may be due to the virtual format of the intervention, lack of efficacy of MBM interventions on anxiety, or other extraneous factors not accounted for. It is possible that the constant, high levels of uncertainty and disruption to daily living with no clear resolution resulted in anxiety refractory to non-psychiatric interventions such as MBM [30]. Although there is preliminary evidence to suggest that mobile meditation apps may reduce anxiety symptoms, it is unknown whether this is generalizable to MBM programs, and if such interventions are effective during public health crises such as the COVID-19 pandemic [31,32].

Alternatively, it is possible that the virtual delivery of MBM training is ineffective in reducing anxiety levels in participants, as multiple studies have suggested that MBM training is effective in reducing anxiety [12]. However, these studies used different anxiety assessment scales, as opposed to the DASS-21 used in this study. Additionally, there may be other unknown extraneous factors not accounted for in this study that affected anxiety levels. Further research is needed to elucidate the effect of MBM training on anxiety among program participants, particularly those using virtual delivery methods.

Evaluation for different stress-reducing strategies associated with the COVID-19 pandemic is increasing. Riley et al. assessed the usefulness and feasibility of the 8-week Mindfulness Based Stress Reduction (MBSR) live online course [33]. Participants of this course reported an increased quality of life, reduced perceived loss of control, and increased morale after completion of the course. Additionally, the course was equally attended by the online and in-person cohorts, suggesting an acceptable feasibility of MBSR delivered through an online medium. Although an online MBSR course may increase accessibility of stress-reducing strategies to the public, it is important to note the social inequalities that may arise from relying strictly on virtual delivery [28].

Additionally, the effectiveness of the Mindfulness-Based Stress Reduction (MBSR) program on sleep quality was evaluated in healthcare workers primarily working with the COVID-19 population. The study found that participants that underwent MBSR reported improved sleep measured by subjective quality, latency, and efficiency [12]. Due to the sleep disturbances associated with common mental health disorders, MBSR and other mindful stress-reduction programs may potentially mitigate the utilization of other harmful coping strategies such as substance use.

While the present results show significant reductions in stress and depression when applied to populations affected by COVID-19, it would need to be shown in future studies whether these effects would carry over to other traumatic events, such as natural disasters, mass shootings, and other forms of mass violence and trauma. In addition to these larger events, it would be of interest to examine whether the present intervention would be effective in reducing depression and stress in other more common types of stressful events, such as occupational related stress and depression, stressful events related to one’s personal or social life, or depression and stress from personal isolation. There is certainly a need for more treatments that are both effective and easily accessible.

### Limitations

Some possible limitations of the present study include the overrepresentation of female and white participants, which limit our ability to generalize our findings to other population groups. The study population was 87.2% female, and while there has been limited research on gender differences in response to mindfulness training, a 2018 randomized controlled trial by Kang, et al. showed that there were response differences between male and female adolescents in a school-based mindfulness training program [34]. However, more research is needed to elucidate whether these differences apply to adult populations. Furthermore, there is a documented gender difference in response rates to pharmacologic treatment of depression [35]. Similarly, there was limited demographic reporting, with 34% of participants declining to report their ethnicity. Previously, it has been shown that ethnicity may exert an influence on individuals’ beliefs about depression and the treatment of depression [36]. It is possible that overrepresentation of certain ethnicities could bias the self-reported responses of participants, and potentially positively or negatively affect their beliefs about the efficacy of the present intervention. Additionally, it is important to note that certain ethnicities were disproportionately affected by COVID-19 [37]. This differential impact on specific ethnicities may have further implications for mental health and the effectiveness of these interventions. Further research is needed to investigate the potential effects of COVID-19 infection rates among different ethnicities on mental health outcomes and the response to interventions like MBM. Next, in the absence of the control group, confounding bias in this study is likely. In other words, it is difficult to pinpoint if the changes we observed from pre- to post-periods were due to the intervention itself as opposed to other confounding factors. In this vein, future studies with a control group can be designed to minimize the potential of confounding bias, thereby increasing internal validity. Lastly, there can also be a residual confounding bias in this study due to some variables that were left unmeasured, such as pre-existing mental health conditions, and whether the participants were receiving mental health care/treatment and/or family/social support. Future trials can be designed to account for these variables to minimize the residual confounding.

Furthermore, there was no data regarding the long-term stability of these results, as the final survey was given only a week after the completion of the MBM course. To properly assess the durability of reductions in stress and depression post-MB-based intervention, subsequent studies should include follow-up assessments over an extended period. By addressing these limitations and exploring their potential impact, future research can provide a more comprehensive understanding of the effectiveness of MBM-based interventions and their applicability across diverse populations, including different ethnicities and genders.

## 5. Conclusions

The COVID-19 pandemic exacerbated existing mental health disorders prevalent in the general population such as anxiety, depression, and substance abuse. Among those most affected in the general populace were healthcare providers and frontline workers. Techniques previously used to manage anxiety and depression have included integrative medicine modalities, such as mind-body medicine. This study investigated how mind-body medicine interventions decreased anxiety and depression and improved quality of life in University of Nevada and Las Vegas healthcare providers, faculty, and students. The results showed a statistically significant decrease in levels of overall stress and depression and an increase in quality of life. However, there was no difference in anxiety, compassion satisfaction, burnout, secondary traumatic stress, and social connectedness levels. This study demonstrated that mind-body medicine was an effective tool during a global crisis and shows potential for use in other public crisis settings such as mass shootings and natural disasters.

## Figures and Tables

**Table 1:** Variables and measures of the survey instruments used in this study.

Survey Tools	Subscales	Number of Items/Scale	Cut-Offs	Measurement	Cronbach’s Alpha
DASS-21 (Total items = 21)	Depression	7 items on 4-point Likert scale	Normal depression = 0–9 Mild depression = 10–13 Moderate depression = 14–20 Severe depression = 21–27 Extremely severe depression = 28+	Categorical The sum score can also be used as the continuous variable.	0.83
	Anxiety	7 items on 4-point Likert scale	Normal anxiety = 0–7 Mild anxiety = 8–9 Moderate anxiety = 10–14 Severe anxiety = 15–19 Extremely severe anxiety = 20+	Categorical The sum score can also be used as the continuous variable.	0.74
	Stress	7 items on 4-point Likert scale	Normal stress = 0–14 Mild stress = 15–18 Moderate stress = 19–25 Severe anxiety = 26–33 Extremely severe anxiety = 34+	Categorical The sum score can also be used as the continuous variable.	0.87
ProQoL Scale (30 items on 5-point Likert scale)	Compassion Satisfaction	10 items on 5-point Likert scale (items 3, 6, 12, 16, 18, 20, 22, 24, 27, 30)	High range corresponds to a good deal of professional satisfaction	Continuous/Numeric	0.84–0.90
	Burnout	10 items on 5-point Likert scale (items 1, 4, 8, 10, 15, 17, 19, 21, 26, 29)	Higher score represents the higher risk of burnout	Continuous/Numeric	-
	Secondary Traumatic Stress	10 items on 5-point Likert scale (items 2, 5, 7, 9, 11, 13, 14, 23, 25, 28)	Higher score represents the higher risk of secondary traumatic stress	Continuous/Numeric	--
SCS		20 items on 6-point Likert scale		Continuous/Numeric	

DASS: Depression, Anxiety, and Stress Scale; ProQoL: Professional Quality of Life Scale; SCS: Social Connectivity Scale.

**Table 2:** Demographic characteristics of study population (n = 94).

Variable	Sample Statistics	95% CI
Age (Median ± IQR ^1^)	45 ± 20	-
Gender, n (%)		
Female	82 (87.2)	0.78, 0.93
Male	12 (12.8)	6.7, 21.2
Ethnicity, n (%)		
Hispanic	4 (4.3)	1.1, 10.5
Non-Hispanic	58 (61.7)	51.1, 71.5
Not reported	32 (34.0)	24.5, 44.5
Race, n (%)		
White	49 (52.1)	41.5, 62.5
Non-white	9 (8.5)	9.5, 17.4
Not reported	36 (38.3)	28.5, 48.9
Role, n (%)		
Staff	61 (64.9)	54.4, 74.4
Community members	33 (35.1)	25.5, 45.6

^1^ IQR = Interquartile range.

**Table 3:** Comparing summary statistics for the psychological outcomes and other measures among program participants at pre and post intervention period.

Outcome	Pre-Program		Post-Program		Mean Difference	T-Statistics	Effect Size (Cohen’s d)	*p* Value
	Mean	S.D.	Mean	S.D.				
Depression	5.60	4.72	2.37	2.77	3.22	5.811	0.599	**<0.001**
Anxiety	5.38	4.89	4.74	5.54	0.64	0.891	0.092	0.2
Stress	10.3	6.69	2.82	1.30	7.45	9.90	1.022	**<0.001**
Overall score (0–120)	21.3	13.89	5.64	4.89	15.62	10.23	1.055	**<0.001**
Professional quality of life	80.89	7.57	82.97	8.07	−2.074	−1.894	−0.195	**0.03**
Compassion satisfaction	40.38	5.02	39.70	5.01	0.68	0.963	0.099	0.2
Burnout	21.94	4.92	23.12	5.22	−1.181	−1.520	−0.157	0.07
Secondary Traumatic Stress	24.79	5.25	24.33	5.39	0.46	−2.176	−0.224	0.2
Social connectedness	73.53	6.33	73.17	5.81	0.362	0.435	0.045	0.3

S.D. = Standard Deviation; DASS scores have been standardized. Significant *p* values are bolded in the table.

**Table 4:** Correlation between psychological outcomes and professional quality of life.

Variables	Depression	Anxiety	Stress	Compassion Satisfaction	Burnout	Secondary Traumatic Stress
Depression	-	0.430 **	0.562 **	−0.247 **	0.211 **	−0.002
Anxiety	0.430 **	-	0.649 **	0.015	0.085	0.173 **
Stress	0.562 **	0.649 **	-	−0.107	0.190 **	0.149 *
Compassion Satisfaction	−0.247 **	0.015	−0.107	-	−0.689 **	−0.104
Burnout	0.211 **	0.085	0.190 **	−0.689 **	-	0.420 **
Secondary traumatic stress	−0.002	0.173 **	0.149 *	−0.104	0.420 **	-

** *p* < 0.01; * *p* < 0.05.

**Table 5:** Comparing levels of depression, anxiety, and stress among program participants pre- and post-program period (N = 94).

Outcome	Pre-Program		Post-Program		Test Statistic	Standard Test Statistic	*p* Value
	N	%	N	%			
	94	50	94	50			-
Depression	-	-	-	-	27.000	−3.554	**<0.001**
Normal	57	60.6	80	85.1			
Mild	23	24.5	7	7.4			
Moderate	10	10.6	5	5.3			
Severe	4	4.3	2	2.1			
Anxiety	-	-	-		51.000	−0.325	0.7
Normal	58	61.7	67	71.3			
Mild	11	11.7	9	9.6			
Moderate	14	14.9	11	11.7			
Severe	11	11.7	7	7.4			
Stress	-	-	-	-	40.000	−2.970	**0.003**
Normal	58	61.7	84	89.4			
Mild	15	16.0	3	3.2			
Moderate	14	14.9	4	4.3			
Severe	7	7.4	3	3.2			

Significant *p* values are bolded.

## Data Availability

The data used in this study can be accessed through the corresponding authors upon reasonable request.
